# Evaluation of Communities of Practice performance developing implementation research to enhance maternal health decision-making in Mexico and Nicaragua

**DOI:** 10.1186/s13012-018-0735-8

**Published:** 2018-03-12

**Authors:** Jacqueline E. Alcalde-Rabanal, Victor M. Becerril-Montekio, Etienne V. Langlois

**Affiliations:** 1 Centre for Health Systems Research, National Institute of Public Health, Av. Universidad 655, Col. Santa María Ahuacatitlán, CP 62100 Cuernavaca, Morelos Mexico; 2 Alliance for Health Policy and Systems Research, World Health Organization, 20 Avenue Appia, 1211 Geneva, Switzerland

**Keywords:** Implementation research, Maternal health, Participatory research, Communities of Practice

## Abstract

**Background:**

Despite the existence of proven solutions to improve maternal health, innovative approaches are still necessary to support the implementation of programs and policies in real-world settings. To address this challenge, we developed a participatory capacity strengthening model based on Communities of Practice (CoPs) of maternal health frontline personnel and decision-makers involved in implementing maternal health programs in Latin America. The activities focused on the conduct and use of implementation research to enhance maternal health decision-making. Our objective is to evaluate the performance of the Communities of Practice in using implementation research to support decision-making in maternal health programs in Mexico and Nicaragua.

**Methods:**

We evaluated the CoPs’ performance using a mixed methods approach. We appraised the performance of CoPs using five criteria: (i) integration of the CoP, (ii) ownership of the methodology, (iii) timely delivery of products, (iv) feedback to decision-makers, and (v) influence on program changes. We also included an assessment of the barriers and facilitators to the conduct and uptake of implementation research findings in maternal health decision-making.

**Results:**

Two CoPs showed “sub-optimal” performance, one was signaled as “needing strengthening,” and three reached “optimal” performance in the use of implementation research to enhance maternal health programs. The relationships between champions, facilitators, and research team were the main internal enabling factor for success. Externally, political and epidemiological environments acted as the main barriers to the performance of CoPs.

**Conclusions:**

Our study highlights the value of involving decision-makers in CoPs, ensuring varied skill sets and profiles of health professionals, as well as maintaining strong and continuous collaborations with researchers. Collaborative approaches and meaningful engagement of decision-makers and researchers are useful in conducting implementation research and promoting the use of evidence to improve maternal health programs in resource-strained settings.

## Background

Important progress has been made throughout the world in maternal health during the last 25 years [1–3]. Nevertheless, the majority of countries that committed to Millennium Development Goal (MDG) 5—reducing maternal mortality ratios by 75% between 1990 and 2015—were not able to achieve that goal [4, 5]. While MDG 5 was not met globally, many critical lessons were learned to support the new Sustainable Development Goals (SDGs) and the implementation of strategies to end preventable maternal mortality by 2030 [6]. An increasing corpus of scientific evidence underlines the complexity of factors influencing the reduction of maternal mortality worldwide [7–11]. Of particular importance among these factors are improvements in the quality and availability of services, training of health personnel [12, 13], responsiveness towards obstetric emergencies [14] including reference and counter-reference systems, and continuum along pregnancy, delivery, and postnatal care services [15, 16]. Despite recent progress to address these issues [17, 18], maternal health policies and programs still face crucial challenges related to the availability of health resources, particularly for disadvantaged groups, the quality of healthcare [8, 19–22], the organization of health services delivery, and maternal health promotion and education.

Addressing these challenges requires planning, development and implementation of maternal health interventions that are properly informed by evidence, and increasing research utilization among decision-makers [23, 24]. In this sense, learning from the important progress made in clinical care following the evidence-based medicine movement, there is increasing support worldwide for evidence-informed health policymaking and health systems strengthening [25–28]. These concepts consider that the complexity of health systems demands a broad approach to evidence as a foundation for decision-making. Yet, implementation of maternal health policies and programs is not easily nor always linked to the use of evidence. This phenomenon often occurs because health services officials must follow operating rules in everyday management of programs, which—albeit supporting standardization of actions—are not regularly updated and enhanced using context-sensitive evidence [29]. Uptake of evidence is also impeded by limited staff competencies, budget constraints, and lack of decision-makers’ interest in research.

Progress remains slow in the implementation and scale-up of proven solutions and effective interventions to lower maternal morbidity and mortality. As such, there is a need to better understand the barriers and facilitators to the implementation of maternal health programs and policies in real-world settings, taking into consideration resource allocation, staffing, and healthcare delivery structures. By scientifically studying the effective delivery of maternal health programs, implementation research aims at optimizing the delivery of existing interventions and improvements in maternal health outcomes.

Implementation research to improve maternal health policies and programs is inevitably influenced by the skills of decision-makers to identify and use evidence, as well as the knowledge derived from the health system workers’ experience [27, 29, 30]. A key to address this challenge is capacity strengthening for the adequate use of information in decision-making for health professionals involved in the provision and management of maternal healthcare services.

As a response, the Alliance for Health Policy and Systems Research, an international partnership hosted by the World Health Organization (WHO), supported an initiative entitled “Capacity strengthening to demand, access and apply implementation research to scale-up maternal health programs for underserved populations in Mexico and Nicaragua.” The overall purpose of the initiative was to foster evidence-informed decision-making towards improved maternal health outcomes [31]. It is aligned with continuing efforts to advance maternal health, including specific targets for maternal healthcare among the Sustainable Development Goals and other global strategies like the Global Strategy for Women’s, Children’s, and Adolescent’s Health 2016–2030 [32].

**Fig. 1:**
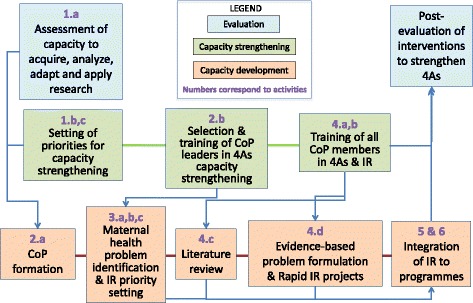
Capacity strengthening model

Mexico is classified as an upper middle-income country with an estimated maternal mortality ratio (MMR) of 38 (34 to 42) deaths per 100,000 live births in 2015 [3, 33], while Nicaragua stands among lower middle-income countries with an estimated MMR of 150 (115 to 195) in 2015 [3, 33]. Striving to address common problems and promote the sustainability of impact, the initiative developed a participatory capacity strengthening model based on Communities of Practice (CoPs) to facilitate primary healthcare units’ management and processes, including input from stakeholders involved in the implementation of maternal health programs. Based on the “four As” approach developed by the Canadian Foundation for Health Improvement (CFHI) [34], the model’s objective was to strengthen individual and institutional capacities to *A*cquire, *A*nalyse, *A*dapt, and *A*pply implementation research, while facilitating its integration in decision-making (Fig. 1). The expected results were executive summaries for decision-makers using a modified version of the 1:3:25 template used by the CFHI [35].

### Communities of Practice for maternal health

We conceive Communities of Practice as groups of people sharing concerns, problems, activities, and interests within their working experience and possessing a deep knowledge and expertise of a specific domain. Previous evidence shows that CoPs are useful in strengthening capacities and fostering a new culture of acting together [36, 37]. In this sense, CoPs provide a setting which is conducive to sharing the lessons and practices of policymakers and health managers in assessing and using scientific evidence. CoPs can also function as interactive interfaces in which researchers and practitioners meet and exchange, thus fostering their personal and professional development [38, 39]. Experiences in different settings demonstrate the advantages of CoPs to support the use of evidence in low- and middle-income countries, particularly for priority-setting approaches to support evidence-informed health system strengthening [40]. CoPs thus have the potential to act as effective and collaborative platforms to strengthen capacities for the use of evidence in health policy and systems decision-making.

While researchers have dealt with the relationship between Communities of Practice, knowledge management and implementation issues in Africa [41], to our knowledge, this is the first time CoPs were established to support the conduct and use of implementation research to improve maternal health programs in Latin America and the Caribbean. An important knowledge gap concerns the contribution of Communities of Practice to health system decision-making and their contribution in light of barriers and facilitators to the use of implementation research to support maternal health programs.

The objective of this paper is to evaluate the implementation and performance of Communities of Practice in the development of implementation research to enhance informed decision-making in maternal health programs in Mexico and Nicaragua, highlighting the barriers and facilitators to the use of evidence.

## Methods

### Phase 1—Development and training of CoPs

**Fig. 2:**
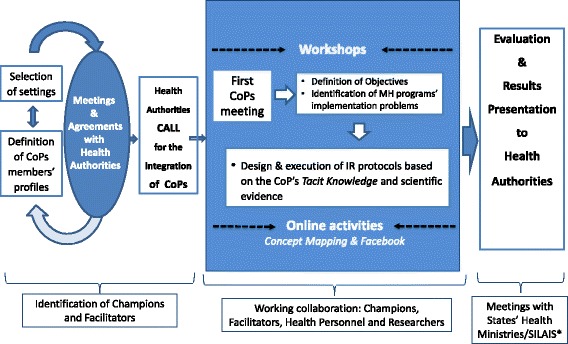
Communities of Practice development, training, and activities. *Health Ministries are the highest level health authorities in each Mexican state and SILAIS in Nicaraguan Departments

We established CoPs in three states of Mexico (Hidalgo, Morelos, and Veracruz) and three departments of Nicaragua (Chontales, Jinotega, and Matagalpa) where existing national maternal health programs were being implemented. The selection of states and departments was based on two main criteria: (a) maternal mortality rates above national averages and (b) presence of health authorities willing to participate in the project. Two research teams, one in Mexico and one in Nicaragua, met with the pertinent health authorities to agree on the objectives of the project, the definition of settings, and the profile of CoPs’ members. Initial contacts with health authorities helped ensure that stakeholders engaged in the CoPs had the authority to perform programmatic changes and improve implementation processes using research findings. The research team documented the context of each CoP emphasizing the most relevant aspects, including sociopolitical, organizational, and epidemiological events. The six CoPs included strategic personnel—health unit directors, maternal health program managers—and tactic frontline staff, including physicians, nurses, and health promoters directly involved in the implementation of maternal health programs. The six CoPs were assigned the same funding to perform the implementation research projects. Development, training, and activities of the six CoPs followed a streamlined process and participation was on a voluntary basis (Fig. 2).

During the first workshop, the research team identified the CoPs’ “policy champions,” namely health personnel with high interest and agency to support the project and communicate its importance to higher level health authorities [39]. We further identified a “facilitator” in each CoP, to act as mediator between the research team, the policy champion, and the CoP members while guiding and promoting members’ participation. We selected facilitators based on their leadership skills and abilities in using information and communication technologies.

Between June 2013 and May 2015, we organized workshops every 2 months with the research team, the policy champions, and the CoP’s facilitators, to provide implementation research training and follow-up on the CoPs’ activities. Concurrently, online activities were organized to strengthen the CoPs’ teamwork through an online platform [42], using a concept mapping process. This method helps conceptualize and give objectivity to the ideas of a group of people on a particular issue. It is particularly useful to identify and prioritize ideas collated in a conceptual framework highlighting their interrelations [42, 43]. We also used social media to convene virtual asynchronic meetings and provide working spaces. Training workshops and online activities focused on conducting literature reviews on implementation problems identified through concept mapping, using research results to inform decision-making, developing implementation research protocols, and presenting implementation research findings. In 2014, four leaders from each CoP attended a 5-day training workshop in Nicaragua focused on the development and execution of implementation research protocols. One more leader per CoP received a full scholarship to attend courses during the Mexico National Institute of Public Health Summer Program on Epidemiology and Public Health.

### Phase 2—Performance evaluation

We assessed the process and performance of the CoPs using a mixed methods evaluation scheme, including an appraisal of the barriers and facilitators to the use of implementation research in maternal health decision-making. In order to appraise the performance of each CoP, we developed an evaluation form based on relevant literature focusing on group collaboration [44] to assess collaborative engagement and decision-making processes [45]. Performance was appraised in relation to five criteria: (a) integration of the CoP, (b) ownership of the methodology, (c) timely delivery of products, (d) feedback to decision-makers, and (e) influence on decision-makers (Table 1). The grid was filled in using data from the field diaries describing the process followed by each CoP and the concept systems platform (Additional file 1).

**Table 1:** CoPs performance assessment criteria. Mexico and Nicaragua, 2013–2015

Criteria	Source of evidence	Value		
		1	3	5
Integration of the CoP Proper communication and consensus mechanisms among CoP members	Concept Systems Global platform	Few members of the CoP communicate among them, while most of them participate randomly in group tasks and discussions	Good communication among all members, but the CoP faces difficulties in finding consensus for action and irregular members’ presence and/or active participation in group tasks and discussions	Good communication among all members facilitating consensus for action and fostering group work and regular members’ presence and/or active participation in the group tasks and discussions
Ownership of the methodology Creative and effective use of implementation research methodology	Results reports presented by CoPs (field diary)	The methodology is deficiently used due to organization problems inside the CoP	The methodology is used and most difficulties are solved	The methodology is used in a swift manner and difficulties are solved with innovative procedures
Timely delivery of products* Capacity to generate and deliver good quality products on time	Results reports presented by CoPs (field diary)	Failure to deliver any of the three main products on time and with satisfactory technical quality	Delivery of the first or second main products with minor technical flaws	Timely delivery of all three main products with satisfactory technical quality
Feedback to decision-makers Presentation of results to maternal health personnel and authorities	Reports on meetings with health authorities (field diary)	The final results of the CoP’s work are only socialized among its members	The final results of the CoP’s work are socialized among peer health personnel or local level health authorities	The final results of the CoP’s work are socialized among local high-level health authorities and health system staff
Influence on program changes Use of evidence generated by the CoP by health authorities	Follow-up of modifications in the maternal health services with decision-makers	The results of the CoP’s work have not been used but its own organization has been useful	The results of the CoP’s work have been used for the implementation of actions to improve the maternal health program in which its members work	The results of the CoP’s work have been used for the implementation of actions to improve the maternal health program in which its members work as well as interventions in other domains

*The three main products were (a) literature review, (b) implementation research protocol, and (c) implementation research report and conclusions

Using this evaluation form, the research team analyzed the information for each CoP and reached a consensus on the corresponding ratings. We then computed the summative score obtained by each CoP as a proxy for their performance. In order to assess different degrees of progress, cut-off points were developed based on terciles of the maximum possible score; the scores were then organized in three ranges 5 to 12 = sub-optimal, 13 to 20 = needs strengthening, and 20 to 25 = optimal.

All members gave informed consent to participate in the CoPs including workshops and online activities. The whole project received authorization from the research and ethics committees of the National Institute of Public Health of Mexico (INSP) and the Centre for Health Research and Studies of the National Autonomous University of Nicaragua, while the implementation research protocols of each CoP were approved by the pertinent health authorities.

## Results

**Fig. 3:**
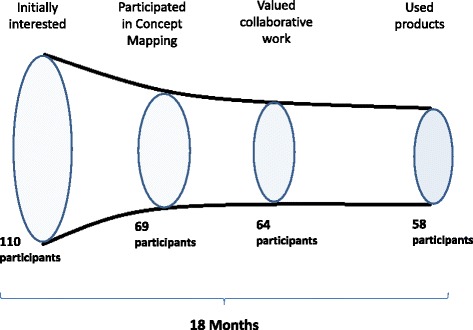
Group dynamics

The final number of participants in the CoPs varied from 17 in Veracruz (Mexico) to 54 in Jinotega (Nicaragua) (Table 2). A total of 326 participants attended the first workshop and 200 participants completed the online concept mapping activities. Attendance to the first workshop followed an invitation by local health authorities, while participation in the subsequent events was voluntary and depended on individual interest and disposition. No pressure was exerted on individuals to participate or not in the project. The process of integration of the CoPs followed the general group integration dynamics, as exemplified by the cases of Hidalgo and Morelos (Mexico) (Fig. 3). The number of professionals who completed the concept mapping process served as the baseline to assess the CoPs’ performance. Taking into account all CoP members at the beginning of the project, 35% of participants were physicians, 37% were nurses, and the remaining 28% were other health personnel or management professionals. By the end of the project, the proportions changed to 43% physicians, 42% nurses, and 15% other.

**Table 2:** Number and nature of health professionals participating in CoPs of Mexico and Nicaragua, 2013–2015

Year	Profession	Mexico				Nicaragua				Total	
		Hidalgo	Morelos	Veracruz	Total	Chontales	Jinotega	Matagalpa	Total	No.	%
2	Physicians	13	21	5	39	16	14	1	31	70	35.0
0	Nurses	11	11	5	27	6	28	12	46	73	36.5
1	Other	4	4	7	15	20	12	10	42	57	28.5
3	Total	28	36	17	81	42	54	23	119	200	100
2	Physicians	13	17	5	35	9	15	1	25	60	42.9
0	Nurses	11	12	5	28	9	12	10	31	59	42.1
1	Other	3	2	7	12	2	5	2	9	21	15.0
5	Total	27	31	17	75	20	32	13	65	140	100

**Fig. 4:**
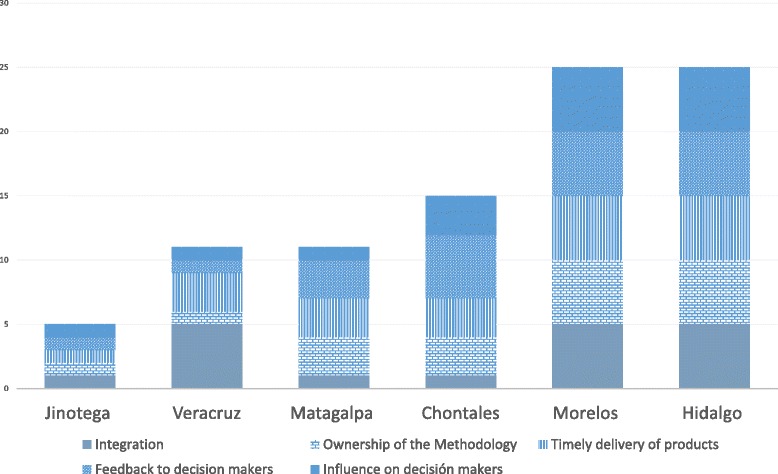
Communities of Practice performance results

Figure 4 shows the rating for each CoP. The CoPs of Veracruz in Mexico, as well as Jinotega and Matagalpa in Nicaragua, barely reached the ratings of 11, 5, and 11 points, respectively. The CoPs of Hidalgo and Morelos (Mexico) obtained the maximum rating of 25 points, while the CoP of Chontales (Nicaragua) achieved 15 points.

### Integration of the CoP

The CoPs of Chontales (Nicaragua) and Hidalgo and Morelos (Mexico) obtained the highest scores. The CoP leaders (champion, facilitator) supported consensus decision-making among participants using available communication mechanisms and motivating active participation. These CoPs created specific WhatsApp groups to support rapid communication among their members. They used social media to monitor progress in different tasks, develop meeting agendas, and stimulate exchanges whenever problems arose. Based on the establishment of horizontal and collaborative relationships among health personnel, health authorities, and the research team, ideas and proposals were regularly discussed and the facilitators triggered consensus building within the group. 

The other three CoPs showed lack of organization among facilitators and champions. This caused delays in the completion of tasks and gradually discouraged participants. There was random attendance to workshops and CoPs did not work as cohesive groups. Most of the time, the tasks were accomplished by the facilitator and/or a few CoP members and lacked general consensus.

### Ownership of the methodology

The capacity strengthening model was set up to train the CoPs on the conduct of implementation research and the use of research findings to inform decision-making. Throughout the project implementation, the individual CoPs either followed strictly, adapted, or were unable to incorporate implementation research methods in their activities. For instance, while the CoP of Morelos respected the proposed literature review process, those of Hidalgo and Matagalpa modified the methods according to the availability of their members. These three CoPs were able to complete the literature reviews according to the established criteria: number of reviewed articles related to the research objectives, identification of main concepts, and final report. On the other hand, the CoPs of Veracruz and Jinotega were unable to follow or adapt the project’s methodology. The CoPs of Matagalpa and Chontales were able to integrate some methodological components, but failed to fully reach the intended goals.

### Timely delivery of outputs

#### Output 1—Literature review

Each CoP carried out a literature review on priority implementation challenges for their maternal health program, based on the outcomes of the concept mapping exercise. The CoPs of Hidalgo and Morelos delivered complete literature review reports. The CoPs of Jinotega, Chontales, Veracruz, and Matagalpa failed to deliver adequate review results to support their program work.

#### Output 2—Implementation research protocols

The three Mexican CoPs developed research protocols to tackle specific implementation problems based on clear objectives and research questions, together with quantitative and qualitative methods suitable to address them. In Veracruz, the CoP delivered the protocol with a 4-month delay and following uneven participation of its members. In Hidalgo and Morelos, all members of the CoPs actively worked on the implementation research protocols and data collection instruments. The CoPs in Chontales and Matagalpa (Nicaragua) also delivered their protocols and instruments, albeit only a small number of participants contributed to the process in Chontales. Table 3 summarizes the issues addressed by the six implementation research protocols.

**Table 3:** Titles of the six protocols developed by CoPs of Mexico and Nicaragua, 2013–2015

Community of practice	Protocol titles
Chontales	Compliance with prenatal healthcare quality norms in the family and communitarian health units of El Ayote and Nueva Guinea in the SILAIS of Chontales
Hidalgo	Compliance with the NOM-007-SSA2-2010 concerning prenatal control in the Health Jurisdiction of Tula, Hidalgo, in 2014
Matagalpa 1	Failures in the response to risk factors of pregnancy by prenatal control health personnel in the Salud Trinidad Guevara Health Center, Matagalpa
Matagalpa 2	Assessment of the implementation of the *Plan Parto* strategy for the reduction of maternal and perinatal mortality in the San José Health Center, municipality of Matigas, in 2014
Morelos	Assessment of the implementation of the Alarm Signs Identification and Complications Prevention Workshops addressed to pregnant women in Morelos in 2014
Veracruz	Compliance with the Prenatal Control Clinical Practice Guide in health centers of Health Jurisdiction VIII of Veracruz in 2014

#### Output 3—Implementation research results and conclusions

Each CoP was committed to present an executive summary of the main findings including key recommendations informed by the implementation research results. Figure 3 shows that five out of six CoPs were successful in delivering this deliverable. Table 4 offers a summary of the main findings and recommendations of the implementation research projects finalized by the CoPs.

**Table 4:** Main findings and recommendations of the CoP implementation research projects. Mexico and Nicaragua, 2013–2015

Community of practice	Main findings	Main recommendations
Chontales	Almost 50% of the personnel is unaware of the need to measure the nutritional level based on maternal weight increase	Organize workshops to strengthen the personnel’s knowledge on this subject
	Most health units have all necessary equipment but 65% of them lack pinard stethoscopes	Assure all health units have these stethoscopes as the pregnant women healthcare norms demand to do
	Regarding laboratory inputs over 90% of the health units lack such tests as rapid plasma reagase, HIV, and toxotest	Regularly check for laboratory inputs in relation to geographical localization of health units
	The main pregnancy alarm signs explained during prenatal consultations were bleeding, headache and abortion	Review the pregnancy alarm signs presented to women to include counseling on edema, particularly in rural communities
Hidalgo	Only 35% of the analyzed medical records satisfactorily comply with the registration of at least 80% of the required information	Develop sensitization activities for prenatal healthcare personnel on the importance of correctly filling in medical records; regularly supervise prenatal care medical records and offer support to the personnel to improve them
	Over 60% of the evaluated personnel responsible for antenatal care do not know how to face obstetric emergencies	Train all personnel on essential actions to face obstetric emergencies and draw flowcharts of these actions to strengthen training
	53% of the personnel consider that the norm requires updating	Organize workshops and meetings with experts to generate evidence and promote the norm’s updating at the national level
Matagalpa 1	Over 50% of the personnel knows the risk classification according to the norms but cannot distinguish potential from real risks; only 23% recognize low risk	Knowledge of pregnancy risk factors must be improved to include clear concepts’ classification and how to handle them properly
	Clinical records show deficient noting of information, particularly for proteinuria (74%), Streptococcus B (67%), multiple antecedents (69%), last registered weight (71%), toxoplasmosis (75%), fetal heart rhythm, and others	Health personnel must be regularly trained and assessed to guarantee the correct use and filling in of clinical records from the very first antenatal care consultation
	Deficient coordination between primary care and the other levels; 53% of the patients were not correctly referred to another level	Promote good coordination between the different levels of care to assure the quality of maternal healthcare.
	There is no plan for risk management, nor specific actions for the personnel to implement the risk identification strategy other than the census of pregnant women	A special sheet must be included to note the main actions of risk management for each pregnant woman
Matagalpa 2	Among pregnant women 37% do not understand the concept and advantages of the *Plan Parto* and 28% do not recognize its importance	Organize information workshops on the *Plan Parto* in communities according to the educational level of the population
	78% of the community leaders understand the concept of the *Plan Parto*, but around 45% do not recognize its advantages and importance	Train community leaders and midwives to assure they have better knowledge of the *Plan Parto* and can thus help the community
	Almost 90% of the community leaders say there are no transport brigades for the pregnant women	Work with the municipality and the community to create transport brigades for the zone taking turns to support women suffering pregnancy risks
Morelos	46% of the interviewed pregnant beneficiaries of Oportunidades do not thoroughly identify pregnancy alarm signs, while only 50% correctly recognize the need to look for medical assistance when these signs are present	Use all available means to improve the quality of the Self-healthcare workshops Make sure that complete information on pregnancy alarm signs and the Security Plan are provided in all medical visits, nursery or health promotion interventions Periodically assess and strengthen pregnant women’s knowledge on alarm signs and the Security Plan
	There is general consensus on the lack of adequate physical spaces and didactic material for the self-healthcare workshops	Create, adapt, or simply assign the appropriate places for workshops Guarantee the availability of all necessary didactic material
	Non-beneficiaries of Oportunidades pregnant women in a control group show worse knowledge of pregnancy alarm signs than beneficiaries	Accept and promote the participation in the self-healthcare workshops of all women attending prenatal control visits in the health centers

### Feedback to decision-makers

The CoPs of Chontales, Hidalgo, and Morelos met with their regional health authorities and presented the main implementation research findings and recommendations. The decision-makers acknowledged the empirical reports and shared them with their staff, with a view of assessing the recommendations to improve implementation and scale-up of maternal health programs.

The CoPs of Jinotega and Veracruz did not present any results to the state’s health authorities, as the implementation research projects were not completed. Finally, the two separate groups of the Matagalpa CoP were not able to engage and share results with the corresponding health authorities.

### Influence on decision-makers

In the state of Hidalgo, the implementation research shed light on poor health personnel’s compliance to national guidelines and norms in antenatal care. After presentation of the findings from the CoP, the health ministry designed and implemented initiatives aiming to improve adherence to antenatal care guidance and standards in primary healthcare facilities. In Morelos, the implementation research showed strong acceptability and feasibility of self-healthcare workshops taught to pregnant women. As such, the health ministry used the findings to adapt health promotion and education interventions targeting pregnant women, in order to improve their response when facing potential maternal health complications. Even when some CoPs did not have a very good performance, the activities and relationships created by the improvement approach had a positive influence by creating local initiatives to improve the implementation of maternal health programs.

## Discussion

In Mexico and Nicaragua, the CoP initiative was set up to strengthen the capacities of maternal health program personnel to generate and use implementation research as important evidence for public health decision-making [22]. The research process was collaboratively conducted by health staff and the research team and embedded in health services management. As such, the process can be considered a case of “embedded research,” as researchers actively collaborated with maternal health programs staff and health authorities, whose role was central in the implementation research projects [46, 47].

Our evaluation suggests that three CoPs performed sub-optimally, while three others showed good integration and ownership of methods. The performing CoPs were thus able to generate relevant implementation evidence as a basis for recommendations to enhance maternal health programs.

Out of the six CoPs, two were able to support decision-makers in addressing newly identified impediments to implementation. Furthermore, the high performing CoPs provide insights on the enabling factors conditioning success of such collaborative platforms. One facilitator is to start with a direct contact with high-level health authorities in each setting. Another critical condition for success is the formation of a collaborative group of actors, champion, facilitator, and research team, working in a participatory fashion.

Effective champions are people who act as interlocutors with authorities to ensure institutional support, while maintaining good horizontal relationships with the members of the CoP. Similarly, CoP facilitators who effectively fulfill their role are recognized by the community as peers with charismatic leadership and organizational capacities and possessing a deep knowledge of the institutional context. These facilitators recognized the importance of collaboration and close contact with the CoP members, as well as regular communication with the champion and the research team. The dynamic of the three actors is key to create synergies and stimulate collaborative work among all members of the CoP.

The integration of the CoP and trust among its members, including the facilitator and champion, were crucial in empowering stakeholders and developing a sense of ownership on the implementation research findings generated. Similarly, strong and continuous relationship-building between the research team, facilitators, champions, and health authorities in the generation of evidence contributed to strengthening the value of evidence to inform maternal health decision-making. The co-production of implementation research findings by health personnel through an embedded research approach also stimulated the context sensitivity and relevance of research for health authorities. Simultaneously, the research team ensured the methodological rigor and scientific quality of the evidence produced. In that regard, our study echoes previous experience speaking to the relevance and potential impact of implementation research embedded in real-world health system settings, including in Latin America and the Caribbean [47, 48].

Another internal factor to consider is the way in which information and communication technologies were intended to be and were actually used by the CoPs. In spite of the generalization of mobile devices and social networks, the difficulty of using them for sustained and in-depth working arrangements became evident during the first months of the project. In fact, the nature of social networks, particularly of *Facebook* groups and pages, proved to be suitable for short, direct communication. But the exchange and generation of deeper content was not always facilitated or promoted by such platforms. Hyperlinks and short messages with clear-cut information travel efficiently on social media, but the interface is not conducive to deeper discussions and evidence-informed exchanges. As a result, the project changed its scope and relied more on live workshops where CoP members met physically to discuss, reflect, and mutually strengthen their capacities.

Externally, various political and epidemiological challenges as well as constant changes among health authorities affected the CoPs’ activities. The CoP of Veracruz struggled with constant political turmoil and turnover of health officials. The dengue and chikungunya epidemics in Nicaragua, and in a less important manner in Veracruz, inevitably diverted the CoPs from their original objectives. Nevertheless, the CoP proved to be an important organizational tool in the fight against these epidemics in Nicaragua, which is an unexpected and encouraging positive externality [22].

Important obstacles to the performance of the CoPs conversely include the lack of direct contact with the highest level health authorities at the onset of the project and the failure to establish a good and regular communication among the three agents of the triad. When the champion or the facilitator was institutionally unable to devote the necessary time to the project or not sufficiently committed to the CoP, the community showed a clear lack of unity. In such circumstances, fewer members had important responsibilities and were responding in timely or effective manners.

Among the strengths of this study, we highlight the combination of tacit and explicit knowledge, the participation of diverse profiles of health staff and decision-makers, and the focus on a specific and relevant topic, i.e., maternal health programmatic implementation. Limitations of this study include an analysis based on a small number of CoPs and follow-up of CoP activities hindered by distance and technological challenges. We must also consider that the way in which CoPs were organized largely conformed to the particularities of Latin American countries, introduced elsewhere [40, 49].

The CoP model proves to be a useful approach to engage health services professionals in the co-development of implementation research to enhance maternal health programs. The model aims to sensitize health services staff on the value and usefulness of implementation research, as well as strengthen their capacities in generating evidence to improve implementation of health interventions. We thus argue for greater co-production of implementation science through collaborative approaches between research professionals and decision-makers. Such efforts can be informed by context-sensitive guidance for health professionals interested in collaborative models in health systems research, including implementation research [50].

## Conclusions

Communities of Practice are relevant platforms to conduct implementation research with the active participation of stakeholders embedded within health systems. This strategy can help bridge the gap between research and action, by generating evidence to improve the implementation of health programs. The role of specialized research personnel collaborating in the CoPs is crucial to uphold scientific validity and ensure technical feasibility. At the same time, engagement of decision-making authorities is essential to ensure political feasibility and participation of frontline health personnel is crucial for operative improvements. Effective functioning of CoPs is also made possible by a collaborative approach in which trust and engagement of peers are essential. Communities of Practice have the potential to stimulate greater integration of decision-makers in real-world research, thus providing value to their tacit knowledge and stimulating a culture of evidence to inform decision-making.

While more research is needed to inform CoP functioning in various health system settings, this study suggests that engagement and mutual support between healthcare staff, decision-makers, and researchers in low- and middle-income countries is a useful approach to co-produce evidence and improve health program implementation and health system strengthening.

## Additional file

Additional file 1: Report template for meetings with CoPs. (DOCX 14 kb)

## Abbreviations

CoP, CoPs: Community of Practice, Communities of Practice
MMR: Maternal mortality ratio
SDGs: Sustainable Development Goals
