# Supplementary material for: Evaluation of Communities of Practice performance developing implementation research to enhance maternal health decision-making in Mexico and Nicaragua
Source: Implement Sci. 2018 Mar 12;13:41. doi: 10.1186/s13012-018-0735-8 (PMC5848447; doi:10.1186/s13012-018-0735-8)
Supplement: Supplementary file 1 — Report template for meetings with CoPs. (DOCX 14 kb) [file 13012_2018_735_MOESM1_ESM.docx]

Annex 1

**Meetings with CoPs Report**

Date ………/………/………

Resercher’s name ……………………………………………………. CoP / Location …………………………

1. **CoP’s Dynamics**
2. Number of participants in the meeting.
3. Describe the participation of the champion and facilitator.
4. Describe the participation of the CoP’s members (communication, relation among them, discussions, conflicts, consensus process).
5. How is the CoP using the proposed methodology?

Can you see any changes or innovation introduced in the methodology by the CoP?

What is the CoP’s opinion about the methodology?

1. According to the particular stage, describe de progress attained by the CoP in
   1. The literature review
   2. The implementation research protocol
   3. The implementation research report
2. Has the CoP faced any problems in the development of this particular stage? Please describe them.
3. Did you notice something unusual or exceptional in this meeting?
4. Describe any particular strengths / weaknesses of the CoP identified during this meeting.
5. **Use of information**
6. How is the CoPs sharing the information gathered by the research? Please give a detailed description of this.
7. Describe any changes or modifications that CoP members propose to improve the program based on the information of their research.
